# How great thou ART: biomechanical properties of oocytes and embryos as indicators of quality in assisted reproductive technologies

**DOI:** 10.3389/fcell.2024.1342905

**Published:** 2024-02-15

**Authors:** Monika Fluks, Rebecca Collier, Agnieszka Walewska, Alexander W. Bruce, Anna Ajduk

**Affiliations:** ^1^ Department of Embryology, Institute of Developmental Biology and Biomedical Sciences, Faculty of Biology, University of Warsaw, Warsaw, Poland; ^2^ Department of Molecular Biology and Genetics, Faculty of Science, University of South Bohemia in České Budějovice, České Budějovice, Czechia

**Keywords:** oocyte, embryo, mouse, preimplantation development, biomechanics, cytoskeleton, quality assessment, assisted reproductive technologies

## Abstract

Assisted Reproductive Technologies (ART) have revolutionized infertility treatment and animal breeding, but their success largely depends on selecting high-quality oocytes for fertilization and embryos for transfer. During preimplantation development, embryos undergo complex morphogenetic processes, such as compaction and cavitation, driven by cellular forces dependent on cytoskeletal dynamics and cell-cell interactions. These processes are pivotal in dictating an embryo’s capacity to implant and progress to full-term development. Hence, a comprehensive grasp of the biomechanical attributes characterizing healthy oocytes and embryos is essential for selecting those with higher developmental potential. Various noninvasive techniques have emerged as valuable tools for assessing biomechanical properties without disturbing the oocyte or embryo physiological state, including morphokinetics, analysis of cytoplasmic movement velocity, or quantification of cortical tension and elasticity using microaspiration. By shedding light on the cytoskeletal processes involved in chromosome segregation, cytokinesis, cellular trafficking, and cell adhesion, underlying oogenesis, and embryonic development, this review explores the significance of embryo biomechanics in ART and its potential implications for improving clinical IVF outcomes, offering valuable insights and research directions to enhance oocyte and embryo selection procedures.

## 1 Introduction

Infertility has been recognized by the World Health Organization (WHO) as a disease and a global public health issue since 2013, following 2 decades of research into its personal and social consequences suffered by those affected (Fidler, 1999; Daar and Merali, 2002; Inhorn et al., 2002; Macaluso et al., 2010; Lemoine and Ravitsky, 2013; Rouchou, 2013; Gimenes et al., 2014; Nik Hazlina et al., 2022). It is estimated that it affects around 10% of couples of reproductive age (Vander Borght and Wyns, 2018), and even 15% of all women (Sadecki et al., 2022).

Assisted Reproductive Technologies (ART), especially *in vitro* fertilization (IVF), have become one of the most important procedures for treating infertility. Although there have been great advancements in IVF procedures over the years, the live birth rate per ART cycle remains low, especially for patients in advanced maternal age. While women below 35 may be expecting, depending on the reporting institution, about 33%/40%/18% success rate, women over 40 face only about 29%/15%/9% chance to give birth (according to Human Fertilisation & Embryology Authority [HEFA], 2021; Society for Assisted Reproductive Technology [SART], 2022; European Society of Human Reproduction and Embryology [ESHRE], 2023, respectively). Consequently, many couples must undergo several IVF cycles to succeed. This brings additional health risks for women, resulting in their psychological and emotional distress (especially in societies where infertility and ART are stigmatized), and further limits ART accessibility due to economic barriers (Daar and Merali, 2002; Cui, 2010; Macaluso et al., 2010; Rouchou, 2013; Nik Hazlina et al., 2022).

Additionally, IVF is an important method in livestock breeding programs (Silber et al., 2013; Sirard, 2018), and the production of phenotypically valuable livestock (Hansen, 2014), which has been a steadily growing area of commerce (Blondin, 2017; Moore and Hasler, 2017; Sanches et al., 2019; Viana, 2019). IVF is also used as a means of overcoming the significant challenges of managing small, isolated populations in zoos (Herrick, 2019) and the preservation of endangered species (Saragusty et al., 2016; Hildebrandt et al., 2018; Kochan et al., 2019; Lanyon and Burgess, 2019).

IVF is increasingly often accompanied by other procedures, such as *in vitro* oocyte maturation and cryopreservation of gametes and embryos. Around 25% of female chemotherapy-treated patients, before the age of 30, develop acute ovarian failure or premature menopause (Letourneau et al., 2012) and the risk reaches 40% for women under 40 and more than 80% for women over 40 (Rosenberg and Partridge, 2013). A strategy to preserve fertility in certain groups of female cancer patients is the cryopreservation of ovarian follicles ahead of the oncological treatment and *in vitro* maturation of oocytes before fertilization after recovery. This approach may also be applied to women with polycystic ovary syndrome, a group of patients with a high risk of ovarian hyperstimulation syndrome (Walls et al., 2015). Both oocyte and sperm cryopreservation are a fertility conservation option for transgender individuals undergoing hormone replacement therapy and genital reconstructive surgery (De Roo et al., 2016) and serve as an efficient banking method of gametes and embryos for infertility patients (Di Santo et al., 2012; Liang and Motan, 2016) and donors (Lindheim and Klock, 2018; Mignini Renzini et al., 2021). *In vitro* maturation and cryopreservation are also widely used tools in assisted reproduction of domestic and endangered animals (Gandolfi and Brevini, 2010; Hildebrandt et al., 2018; Sjunnesson, 2020).

The efficiency of IVF can be raised by transferring multiple embryos in a single cycle, but it can result in multiple pregnancies and, as a consequence, in serious health complications for mothers and their offspring (Ombelet et al., 2005; Skora and Frankfurter, 2012). Many clinics have therefore introduced elective single embryo transfers (eSET), according to guidelines of good clinical practice. Consequently, scientists and the medical industry are urged to develop novel and reliable methods to select high-quality embryos for transfer. Thus, protocols for noninvasive assessment of embryo competence are a valuable addition to the IVF procedures. Furthermore, the evaluation of oocyte quality serves the purpose of selecting the most suitable oocytes for fertilization. This becomes especially vital when legal restrictions limit the number of eggs that can be fertilized (e.g., six in Poland, unless specific medical conditions or age criteria are met). Reliable evaluation processes allow embryologists to personalize IVF treatments for each patient, including considerations such as the logistics of oocyte cryobanking and helping to manage patient expectations. Equally significant, the outcome of oocyte evaluation can yield supplementary insights that prove valuable in assessing the quality of the resulting embryos.

Plenty of methods for oocyte and embryo selection have been previously proposed (Patrizio et al., 2007; Rienzi et al., 2011; Ajduk and Zernicka-Goetz, 2013; Anagnostopoulou et al., 2022), but their adaptation into a clinical setting has proved challenging, either due to conflicting results or time, personnel, and financial constraints (Ajduk and Zernicka-Goetz, 2013). Current methods used in clinics are primarily based on the assessment of oocyte or embryo morphology, often combined with time-lapse imaging providing extra information on cellular divisions and morphogenetic events occurring during embryo preimplantation development (so called morphokinetics). However, morphology assessment of oocytes is often more informative than predictive (Nikiforov et al., 2022), and in embryos—it allows for excluding low-quality specimens, but not necessarily for indicating those of the highest viability (Gardner and Balaban, 2016). Moreover, this approach is prone to intra- and interobserver bias (Paternot et al., 2009; Bormann et al., 2020). Recently, artificial intelligence algorithms have been explored as a means of enhancing these methods (Zaninovic and Rosenwaks, 2020). However, some scholars point out that this approach still requires proper standardization of methodology (Kragh and Karstoft, 2021). Another technique applied in the evaluation of embryos is preimplantation genetic testing (PGT; Madero et al., 2023). Preimplantation genetic testing for monogenic gene defects (PGT-M) is a well-established method for selecting embryos devoid of disease-related mutations. However, the efficiency and accuracy of preimplantation genetic testing for aneuploidy (PGT-A) and preimplantation genetic testing for structural rearrangements (PGT-SR) remains limited, due to embryonic mosaicism (Popovic et al., 2020; Giuliano et al., 2023). Moreover, all types of preimplantation genetic testing are highly invasive, requiring biopsy of embryonic cells (as a source of genetic material for analysis). Furthermore, PGT-A is controversial due to the lack of unambiguous evidence to support its use (Mastenbroek et al., 2021).

As quality assessment protocols still require improvement, numerous novel methods of quality assessment have been proposed in recent years. One is the metabolic profiling of the embryos, which is achieved by the chemical analysis of spent culture media (Nagy et al., 2009; Zhao et al., 2013; Salmerón et al., 2021). Another technique, fluorescent lifetime imaging microscopy (FLIM) uses the differences in the exponential decay rate of the photon emission of autofluorescent coenzymes NAD(P)H and FAD^2+^ and allows for quantification of their concentration and thus energy metabolism of the embryo (Ma et al., 2019; Venturas et al., 2022; Venturas et al., 2023). This technique, however, uses UV light to excite autofluorescence, raising concerns about its invasiveness. Finally, it is also possible to analyze the chemical composition of oocytes and embryos using coherent anti-Stokes Raman scattering (CARS; Bogliolo et al., 2013; Davidson et al., 2013; Bradley et al., 2016; Jasensky et al., 2016; Ishigaki et al., 2017; Rusciano et al., 2017; Arena et al., 2021). CARS detects the vibrational spectra of biomolecules, depending on the mass of the atoms constituting the molecule and the strength of their respective bonds (Robert, 2009). Although not particularly efficient in identifying proteins, CARS provides a reliable quantitative analysis of lipids (Evans and Xie, 2008; Zumbusch et al., 2013).

Recently, the application of biomechanical quality assessment in ART has been a growing area of research as well. Biomechanical properties of oocytes and embryos reflect the functionality of key cellular components, including cytoskeleton and intracellular junctions. Therefore, examination of the oocyte/embryo biomechanics may provide novel insights into the quality of those intracellular structures, and, in consequence, improve oocyte/embryo evaluation protocols. Many invasive approaches to the analysis of the biomechanical properties of cells have been proposed. Structure of biomechanically relevant cellular components may be studied with fluorescent probes and confocal microscopy (Kölle et al., 2009). On the other hand, confocal microscopy, together with special nanomechanical chips, can be used to assess the intracellular pressure (Gómez-Martínez et al., 2013). The chip comprises of a mechanical sensor and an optical reference area created by two parallel reflecting membranes, separated by a vacuum gap. Waves can only pass through the reference area when they are in resonance with it. By analyzing the reflected light’s intensity, the system can quantify the pressure-induced membrane deflection. Another interesting method is the implementation of magnetically responsive ferrofluid microdroplets that enable highly precise measurements of mechanical properties, such as viscosity in tissues and embryos (Campàs, 2016; Serwane et al., 2017). The viscosity of tissue affects the movement and deformation of the microdroplets under the influence of the magnetic field, allowing for precise quantitative measurements of mechanical properties. While these approaches have shown promise in research, their application in ART is limited due to concerns about their potential to disrupt natural developmental processes. Noninvasive techniques described in this review (Table 1) represent an alternative, yet promising, avenue for gaining deeper insights into the biomechanical aspects of oocytes and embryos during ART, offering valuable contributions to improving clinical outcomes and reproductive health.

**TABLE 1:** Advantages and limitations of noninvasive techniques used in assessment of biomechanical properties of oocytes and embryos.

Technique	Type of information provided	Advantages	Disadvantages
Analysis of cavitation dynamics	- Timing and extent of blastocoel expansion	- Enables estimation of the biomechanical properties of the blastocyst	- Requires *in vitro* culture up to expanded blastocyst stage and periodic exposure to light (time-lapse imaging)
	- Parameters describing amplitude, frequency, and duration of expansion-contraction cycles of the blastocoel	- May utilize standard time-lapse recordings obtained for morphokinetic analysis	- Does not offer information about sub-cellular structures
		- Quantifiable data	
Analysis of cytoplasmic velocity	- Velocity and direction of the cytoplasmic movement	- Simple technique providing biomechanically relevant measurements	- Measurements are very easily disrupted by external movement of the analyzed cells
	- Fast cytoplasmic movements typical for freshly fertilized oocytes precisely mimic the pattern of sperm-induced Ca^2+^ oscillations	- Quantifiable data	**-** Until now has been applied only in experimental setting
Atomic force spectroscopy (AFS)	- Young’s modulus, stiffness, and adhesion force	- Provides precise biomechanical measurements at the nanoscale	- Limited to studies of the *zona pellucida* (making it imprecise for oocyte/embryo properties assessment) or requiring *zona* removal (making it invasive)
		- Quantifiable data	- Expensive setup and maintenance costs
			- Until now has been applied only in experimental setting
Cortical tension (CT) measurements by microaspiration	- Measures the force required to aspirate a portion of the cell, providing information about its surface tension or viscoelastic properties	- Simple technique providing biomechanically relevant measurements	- Limited to studies of the *zona pellucida* (making it imprecise for oocyte/embryo properties assessment) or requiring *zona* removal (making it invasive)
		- Quantifiable data	**-** Until now has been applied only in experimental setting
Harmonic generation microscopy (HGM)	- Imaging of sub-cellular morphology, including metaphase spindles, based on nonlinear optical processes	- Provides 3D sub-cellular structural information	- If light of longer wavelength is used, low spatial resolution limits detailed analysis, for shorter wavelengths—potentially invasive
			- Obtained data is only indirectly related to cellular biomechanics
			- Requires an expensive setup and maintenance
			**-** Until now has been applied only in experimental setting
Optical coherence microscopy (OCM)	- 3D reconstructions of intracellular architecture, including metaphase spindles, based on intrinsic contrasting of back-scattered coherent light	- Offers 3D sub-cellular structural information	- Limited spatial resolution affecting detailed structural analysis
		- Quantifiable data	- Obtained data is only indirectly related to cellular biomechanics
			- Requires a fairly expensive setup
			**-** Until now has been applied only in experimental setting
Polarized light microscopy (PLM)	- Visualization of anisotropic cellular structures, such as metaphase spindles or zona pellucida, based on detection of changes in refractive indices and birefringence	**-** Used in ART on every-day basis	- Obtained data is only indirectly related to cellular biomechanics
		- Simple technique	
		- Quantifiable data	
Quantitative phase imaging (QPI)	- Captures phase shifts and refractive indices using off-axis illumination	- Offers 3D sub-cellular structural information	- Applicability limited to high refractive index structures within cells
	- Quantitative imaging of sub-cellular morphology	- Quantifiable data	- Obtained data is only indirectly related to cellular biomechanics
			**-** Until now has been applied only in experimental setting

## 2 Cytoskeletal functions, dynamics, and alternations in oocytes

The developmental capabilities of mammalian embryos are largely determined by the oocyte cargo (Stitzel and Seydoux, 2007; Li et al., 2010). Although fragmentation of DNA or other deterioration of sperm can diminish an embryo’s potency, the oocyte contributes the vast majority of the cytoplasmic contents: cytoskeleton components essential for a multitude of inter- and intracellular processes, mitochondria providing energy to the embryo, lipid droplets supplying metabolic reserves, and maternal mRNA and proteins accumulated during oocyte growth, required as a guiding template before embryonic genome activation. Identifying features of a high-quality oocyte can therefore facilitate ART procedures.

Studies have shown that the biomechanical properties of mammalian oocytes reflect their developmental competence (Ebner et al., 2003; Liu et al., 2010). Whilst these properties stem partially from the *zona pellucida*’s mechanical characteristics, they predominantly depend on cellular biomechanics. The zona may harden as a result of cortical granule exocytosis at fertilization (the main element of the polyspermy block; Murayama et al., 2006; Shen et al., 2019), but also, as shown in the mouse, during premature cortical granule exocytosis in the *in vitro* matured and vitrified oocytes (Carroll et al., 1990; Ducibella et al., 1990). Premature zona hardening inhibits *in vitro* fertilization via gamete co-incubation, as sperm cannot penetrate the zona (Carroll et al., 1990). Conversely, cellular mechanics, *i.e*., elastic (ability to resist deformation) and plastic (ability to undergo permanent deformation) behavior of cells, reflect their cytoskeletal functionality (Larson et al., 2010; Chaigne et al., 2013; Chaigne et al., 2015; Mackenzie et al., 2016). The cytoskeleton plays a key role in the segregation of chromosomes, cytokinesis, and cellular trafficking, each of which is important for cell cycle progression (Tang, 2012; D’Avino et al., 2015; Prosser and Pelletier, 2017). Microtubules build metaphase spindles, while actin and myosin are required for the spindle positioning and formation of the cytokinetic contraction ring (Schuh and Ellenberg, 2008). Disturbances in the function of these components can result in aneuploidy, which has detrimental effects on future embryonic development, especially when first appearing in meiosis (McCoy et al., 2023). A functioning actomyosin cytoskeleton is also invaluable during so-called cytoplasmic maturation, a process concurrent with the meiotic maturation of oocytes. Research conducted predominantly on mouse oocytes indicates that during cytoplasmic maturation relocation of organelles occurs. Mitochondria move from the perinuclear region towards the cell periphery (Dalton and Carroll, 2013), the Golgi apparatus fragments and shifts to the center of the gamete (Moreno et al., 2002), and the endoplasmic reticulum gathers in the cortical region (Mehlmann et al., 1995; FitzHarris et al., 2007). These changes in organelle distribution depend on intact actin filaments (Dalton and Carroll, 2013). Simultaneously, dynamic changes in the cytoskeleton itself occur. In mouse oocytes, metaphase I spindle migration is supported by an ARP2/3-dependent thickening of the cortical F-actin meshwork, nucleated by formin-2 (Leader et al., 2002; Dumont et al., 2007; Schuh and Ellenberg, 2008) and Spire 1/2 (Pfender et al., 2011). As the cortical F-actin thickens, myosin-II is excluded from the cortex, leading to its softening (Chaigne et al., 2013). These events are regulated by phosphorylated (active) myosin-II regulatory light chain and phosphorylated ezrin-radixin-moesin complex and coordinated temporally by the MOS-MAPK pathway (Larson et al., 2010; Chaigne et al., 2013; Chaigne et al., 2015). Cortical tension (CT) resulting from the force generated by the actomyosin cytoskeleton must be strictly regulated to allow normal spindle migration and positioning: too low or too high CT both lead to spindle anomalies (Chaigne et al., 2013; Chaigne et al., 2015). As shown for human and mouse oocytes, actin filaments may also infiltrate the meiotic spindle and regulate the correct alignment and segregation of the chromosomes (Mogessie and Schuh, 2017).

Interestingly, Larson and others show that there are some discrepancies in cytoskeleton modifications during meiotic maturation between *in vivo*- and *in vitro*-matured mouse oocytes (Larson et al., 2010). This could be linked to lower developmental capabilities of *in vitro* matured human metaphase II oocytes (Jurema and Nogueira, 2006). The cellular cytoskeleton is often damaged during freezing and thawing procedures as well (Hosu et al., 2008; Hendriks et al., 2015). Various cytoskeletal elements can also be affected by postovulatory aging (*i.e*., the extended period between ovulation and fertilization; reviewed in Miao et al., 2009; Takahashi et al., 2013); in particular, actin distribution and myosin functionality (McGinnis et al., 2015; Mackenzie et al., 2016). Some authors have even suggested that reduced myosin light chain kinase activity in aged mouse oocytes is linked to their susceptibility to parthenogenetic activation, potentially by dysregulation of membrane ion channels (McGinnis et al., 2015; Mackenzie et al., 2016).

Cytoskeletal damage can be caused by reactive oxygen species (ROS; Lord et al., 2013). In somatic cells, proteins damaged by oxidation, such as carbonylated or glycated proteins, accumulate with age and in several pathological states (Stadtman, 1992; Levine, 2002). These proteins are rendered inactive and tend to form large aggregates in the cytoplasm. Similarly, the mammalian germline accumulates these dysfunctional proteins (Hernebring et al., 2006; Haucke et al., 2014), but they are eliminated to some extent during embryo development (Hernebring et al., 2006). Oocytes may carry varying amounts of advanced glycation end (AGE) products and carbonylated or otherwise modified proteins, depending on maternal age and overall female health. Notably, actin is a common target for carbonylation (Aksenov et al., 2001; Soreghan et al., 2003). Mouse oocytes subjected to postovulatory aging or obtained from females of advanced reproductive age feature increased ROS concentrations (Szpila et al., 2019; Czajkowska and Ajduk, 2023). Similarly, vitrification is known to cause oxidative stress in murine, porcine, and human oocytes (reviewed in Tatone et al., 2010). Some studies report that increased ROS levels can be also observed during murine and bovine oocyte *in vitro* maturation (Morado et al., 2009; Xie et al., 2016). It is feasible that carbonylation/glycation of proteins occurs not only in oocytes obtained from old females but also in oocytes otherwise subjected to oxidative stress (Berlett and Stadtman, 1997), including postovulatory aging and vitrification. It can be speculated that these oocytes’ cytoskeletal dysfunction could be, at least in part, caused by the oxidation of cytoskeletal proteins (Mihalas et al., 2018).

Cytoskeletal functionality is intricately linked to the successful progression of meiosis, making it a pivotal factor in oocyte and early embryo development. As a result, the assessment of cytoskeleton quality in these cells, reflected among others by their biomechanical properties, emerges as a valuable and predictive indicator, offering critical insights into the outcomes of IVF procedures, thus enhancing the understanding and potential success of ART.

## 3 Quality assessment of oocytes based on cytoskeletal and biomechanical properties

Some biomarkers indicative of the biomechanical properties, e.g., *zona pellucida* and metaphase spindle, can be observed using polarized light microscopy (PLM; Oldenbourg, 2013; Ajduk and Szkulmowski, 2019). Importantly, zona birefringence also indicates its ability to participate in the acrosome reaction and ability to undergo proper polyspermy block, whereas shape of the metaphase spindle can be associated with the ploidy and the maturation status of the oocyte (Caamaño et al., 2010; Montag et al., 2013; Omidi et al., 2017).

Alas, even PLM does not provide detailed information on inner cell architecture, nor does it have a high in-depth resolution. These limitations might be overcome by harmonic generation microscopy (HGM; Hsieh et al., 2008; Thayil et al., 2011), which obtains contrasts by the sample’s ability to emit photons with half the wavelength of incident light. HGM has been shown to obtain 3D images of microtubules in the spindle (Yu et al., 2014; Sanchez et al., 2019) and myosin heavy-chain B (Mohler et al., 2003). However, HGM is beset by the choice between the imaging depth when using longer excitation wavelengths, which are less harmful to biomaterials, and high spatial resolution when using shorter but more invasive wavelengths (Aghigh et al., 2023). These limitations are shared by optical coherence microscopy (OCM), which can be used for metaphase spindle visualization (Karnowski et al., 2017). In addition, both methods typically require a complex and expensive setup, which might not be readily available in prospective fertility clinics. A simpler solution can perhaps be found in quantitative phase imaging (QPI) techniques, such as holographic microscopy or interferometric phase imaging, which can provide quantitative information about cell morphology based on 3D refractive index distribution (Nguyen et al., 2022). These methods can be used to study cytoskeletal organization and dynamics (Bon et al., 2014), however, to date QPI has mostly been employed to study membranous organelles such as mitochondria, which have a higher refractive index (Sandoz et al., 2019; Salucci et al., 2020).

**FIGURE 1:**
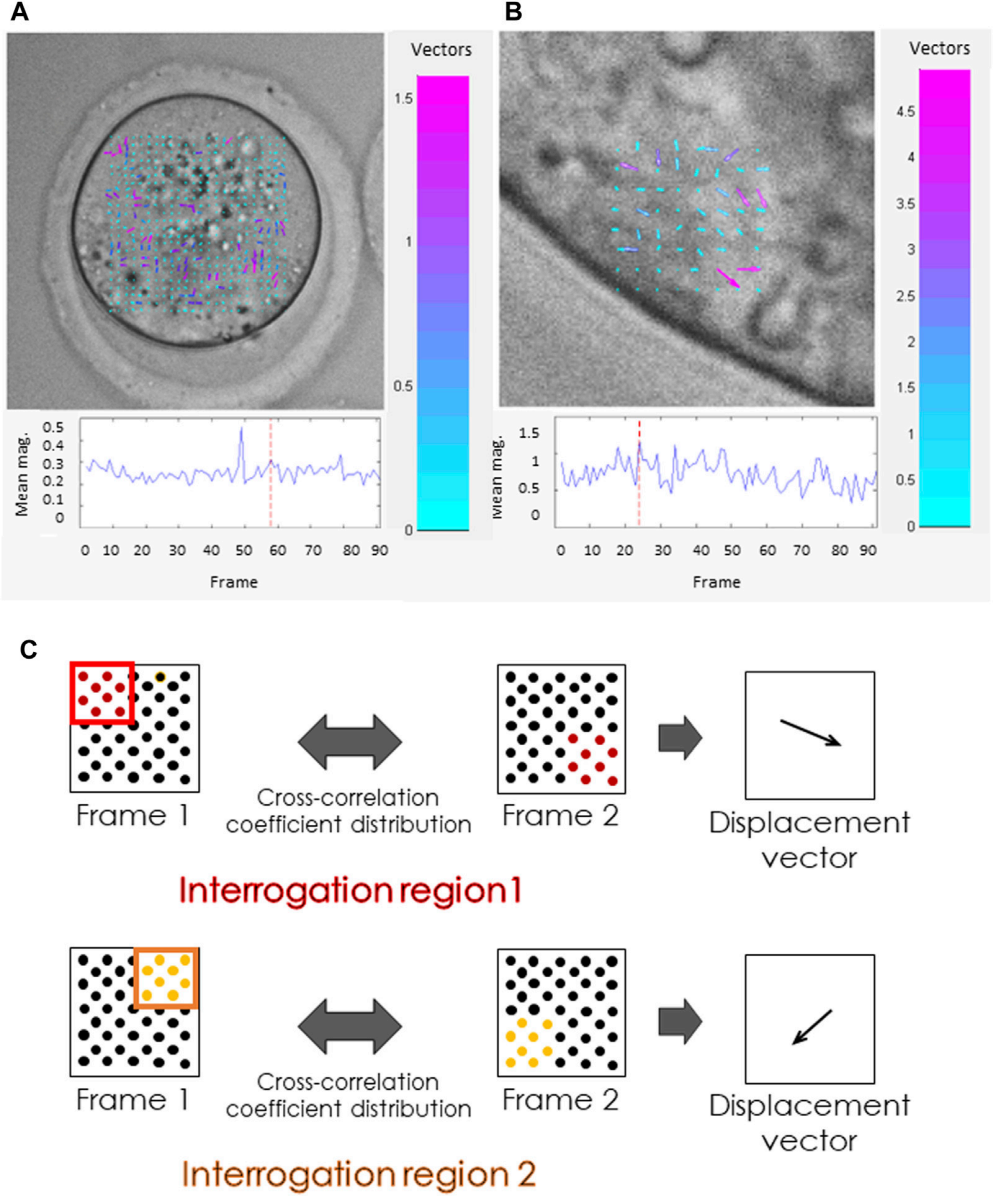
Cytoplasmic movement velocity (CMV) assessment by Particle Image Velocimetry (PIV). **(A, B)** Images from the PIV software used by some of the authors (Ajduk et al., 2011). PIV analysis was conducted for high-resolution time-lapse images of **(A)** mouse metaphase II oocyte, **(B)** polar trophectoderm cell in a mouse blastocyst. Both the length and the color of the vectors visible inside the cells reflect the cytoplasmic velocity. Cyan represents the slowest-moving vectors. Magenta represents the fastest-moving vectors. The graphs (below) show the mean cytoplasmic velocity in the analyzed region over time. The direction of the vectors indicates the direction of cytoplasm displacement between frames. **(C)** Schematic representation of the PIV algorithm. The algorithm divides the images into small interrogation windows, identifying the pattern of pixels in each window, and calculating the displacement of particles between frames. This information is used to generate a map of velocity vectors representing the cytoplasmic flow and to calculate mean cytoplasmic velocity.

Cellular mechanics depend on cytoskeletal function, thus probing oocytes or embryos for their biomechanical characteristics can provide insight into their developmental potential. One such method is the analysis of cytoplasmic velocity. Cytoplasmic velocity measurement combines time-lapse imaging with particle image velocimetry (PIV) analysis. PIV is an algorithm frequently used in fluid dynamics that follows the displacement of bright and dark pixel patterns in consecutive images to establish the velocity and direction of the fluid (cytoplasm) movement (Westerweel, 1997; Figure 1). Studies have demonstrated that cytoplasmic velocity monitored at the time of fertilization reflects the capacity of mouse zygotes to correctly complete preimplantation and full-term development (Ajduk et al., 2011). These movements can also be observed in human zygotes (Swann et al., 2012). Fast directional cytoplasmic movements (the so-called “speed-peaks”) correspond to rhythmic actomyosin-mediated spasms, coincident with fertilization-induced Ca^2+^ oscillations (Ajduk et al., 2011). The mean basal speed represents the average velocity of cytoplasmic movement between speed-peaks, thereby providing information on the functionality of the zygote actomyosin cytoskeleton. For example, a relative decrease in mean basal speed is concurrent with both depolymerization and overstabilization of actin filaments and inhibition of myosin (Ajduk et al., 2011). Moreover, the mean basal speed in freshly fertilized mouse oocytes correlates with the length of the second embryonic cell cycle, the percentage of cells with fragmented nuclei, and the percentage of primitive endoderm cells in the blastocyst (Milewski et al., 2018). The basal velocity of cytoplasmic movement in unfertilized (metaphase II) oocytes is slower than in their fertilized counterparts (Ajduk et al., 2011). Both maternal and postovulatory types of aging are detrimental to the actomyosin cytoskeleton of mammalian, including human, metaphase II oocytes (Pickering et al., 1988; Sun et al., 2012; McGinnis et al., 2015; Mackenzie et al., 2016; Dunkley et al., 2022), which might influence the cytoplasmic dynamics, resulting in lower basal speed in aged oocytes.

Interestingly, the mean basal cytoplasmic speed is also indicative of immature oocyte (so-called GV oocyte) quality (Bui et al., 2017). There are two populations of GV oocytes, which are known to have distinct developmental capabilities: oocytes with surrounded nucleolus (or SN oocytes) that have already transcribed all necessary RNAs and are transcriptionally inactive, and oocytes with non-surrounded nucleolus (or NSN oocytes) that are still transcribing (reviewed in: Tan et al., 2009). These two types of GV oocytes differ in terms of the basal speed at various stages of *in vitro* maturation (Bui et al., 2017).

**FIGURE 2:**
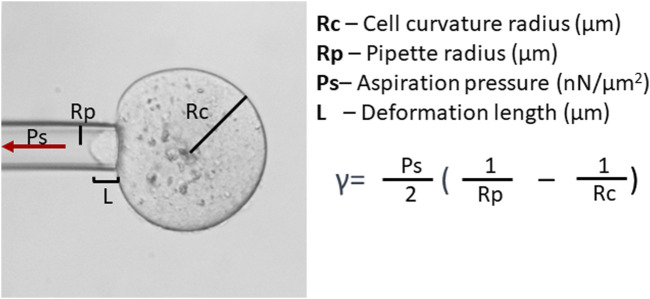
Cortical tension (CT, γ) analysis. CT analysis can be conducted by micropipette aspiration. Assessment of CT requires the measurement of cell curvature radius and aspiration pressure (Ps) when the deformation length (L) becomes equal to the micropipette radius (Rp) and utilizes the Young–Laplace equation.

Assessment of oocyte quality based on the monitoring of actomyosin cytoskeleton-mediated cell mechanics can also utilize techniques such as micropipette aspiration (Mackenzie et al., 2016; Yanez et al., 2016) and indentation (Liu et al., 2012). Applying negative pressure through a micropipette or positive force through a microlever, results in the deformation of the cell allowing for the calculation of the cell’s physical properties, such as CT or elasticity (Figure 2). CT reflects the biochemical and structural features of the oocyte cortex (Larson et al., 2010; Chaigne et al., 2013; Chaigne et al., 2015; Mackenzie et al., 2016) and *zona pellucida* (Khalilian et al., 2010; Shen et al., 2019). Studies on mouse oocytes devoid of *zona pellucida* have shown that CT decreases six-fold during maturation, then increases about 1.6-fold after fertilization (Larson et al., 2010). Also, mature mouse oocytes are polarized, with CT differing 2.5-fold between the stiff cortex over the meiotic spindle (the amicrovillar domain) and the softer, opposing cortex, where the sperm binds (microvillar domain; Larson et al., 2010). The purpose of this asymmetry is unclear. However, *in vitro* matured oocytes have reduced tension in the amicrovillar domain (Larson et al., 2010). Viscoelastic equilibrium in the cortex is essential to achieve asymmetric cytokinesis (Chaigne et al., 2013; Chaigne et al., 2015). This equilibrium is characteristic of a viable oocyte (Yanez et al., 2016). Too elastic or too plastic cortex results in anomalous spindle migration (Chaigne et al., 2013; Chaigne et al., 2015) and lowered developmental competence, likely due to less effective cortical granule release at fertilization, which could lead to polyspermy (Yanez et al., 2016). CT is also reduced in both maternally (Liu et al., 2012) and postovulatory-aged oocytes (Mackenzie et al., 2016). These differences in viscoelastic properties, hence cytoskeletal properties, offer a valuable tool for quality assessment. Methods for testing oocyte and embryo cytoskeletal properties by CT analysis, however, are relatively time-consuming and labor-intensive. Additionally, most protocols presented to date feature the removal of the zona, which may negatively affect overall embryo development (Fan et al., 2022).

**FIGURE 3:**
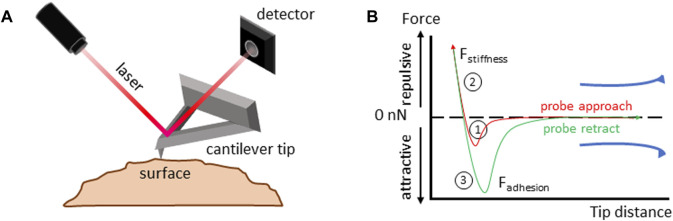
Atomic force spectroscopy. **(A)** Atomic force spectroscopy setup diagram. Details in the main text. **(B)** Force-distance curve analysis to measure the viscoelastic properties of a material. 1) The cantilever tip approaches the sample until it makes contact, and the force at the interaction between the tip and the sample is measured; 2) the tip is further compressed into the sample, deforming its surface, the forces acting on the tip during compression are recorded, allowing for calculation of the surface stiffness; 3) the tip is retracted from the sample surface. Forces acting on the tip as it moves away from the sample are measured allowing for calculation of surface adhesion.

Another method to measure surface forces is atomic force spectroscopy. Here, a sharp tip attached to a cantilever runs over the surface of the sample. The deflection of the cantilever across the sample surface is measured using a laser beam, which is reflected onto a photodetector (Figure 3). The amount of deflection is used to calculate the force exerted on the tip by the sample’s surface (Viljoen et al., 2021). In its noninvasive form, in a similar manner to CT measurements, atomic force spectroscopy is limited to the studies of the *zona pellucida* (Boccaccio et al., 2012; Andolfi et al., 2016; Battistella et al., 2022).

## 4 Molecular basis of embryo biomechanics

The success of *in vitro* fertilization procedures is also contingent on sperm quality and conditions of embryo culture (Chapuis et al., 2017; Colaco and Sakkas, 2018; Consensus Group, 2020). Various molecular mechanisms reflected in changes in biomechanical properties are at play before the blastocyst, the last stage of mammalian preimplantation development, is formed. A blastocyst is built of an inner cell mass (ICM) and an outer trophectoderm (TE) epithelial layer. As the blastocyst expands, ICM cells differentiate into two lineages: the centrally located epiblast (EPI) and the primitive endoderm (PE), also called hypoblast, adjoined to the blastocyst cavity (blastocoel). The TE, on the other hand, differentiates into the polar TE surrounding the ICM and the mural TE surrounding the blastocyst cavity. The ICM that will give rise to the embryo proper (resulting from the EPI) as well as additional extraembryonic membranes (derived from the PE), and the TE will participate in embryo implantation in the uterus and form the future placenta.

Preimplantation development consists of multiple mitotic divisions, which are highly dependent on actin networks (Chaigne et al., 2016). Importantly, zygotic genome activation (ZGA) is connected with maternal protein degradation (Toralova et al., 2020), and considerable changes in cytoskeletal makeup. During embryonic development, the configuration of cells and cell lineages is shaped by the contractile nature of the actomyosin cortex (Murrell et al., 2015; Coravos et al., 2017; Özgüç and Maître, 2020). These cortical contractile forces are essential for the formation of the cleavage furrow during cytokinesis (Fujiwara and Pollard, 1976; Straight et al., 2003; Yamamoto et al., 2021), the movement of cells during migration and maintenance of appropriate cell positioning (Eddy et al., 2000; Tsai et al., 2019), and the withdrawal of cellular blebs (Charras et al., 2006; Taneja and Burnette, 2019). Moreover, changes in actomyosin contractility drive processes such as apical constriction (Martin et al., 2009; Solon et al., 2009) and the restructuring of cell-cell contacts (Bertet et al., 2004; Maître et al., 2012).

Significant biomechanical alterations occur particularly during the compaction of preimplantation embryos, a process that occurs at the 8-cell stage in mice, 16-cell stage in humans, and 32-cell stage in rabbits and cattle (Płusa and Piliszek, 2020). Compaction is accompanied by intra-cellular polarization of blastomeres along the apical-basal axis. In pre-compacted mouse embryos, actomyosin is uniformly distributed in the cellular cortex, whereas during compaction, it accumulates gradually in the apical, contact-free region, and becomes excluded from the cell-cell contact sites (Zhu et al., 2017). Moreover, as the mouse embryo undergoes compaction, the cell adhesion protein, epithelial cadherin (E-cadherin), translocates and becomes phosphorylated at the cell-cell contact sites (Winkel et al., 1990). Additionally, ezrin, a protein responsible for establishing and maintaining microvilli, undergoes phosphorylation and relocates to the contact-free regions of the membranes (Louvet et al., 1996).

Translocation of actomyosin occurring during compaction leads to the progressive increase of the CT on the contact-free interface of the blastomeres. At the same time, junctional E-cadherin keeps actomyosin contractility low at the cell-cell contacts (Maître et al., 2015). As a result, the inner and outer cells of a compacted embryo differ in contractility (Maître et al., 2016). These changes in cellular adhesion and CT are crucial not only for mouse embryo compaction but also for the internalization of the cells that will later form the inner cell mass (ICM; Samarage et al., 2015; Maître et al., 2016).

The biomechanical properties of preimplantation embryos are dynamically regulated by various molecular pathways. The initiation and maintenance of symmetry breaking in a compacting mouse embryo depend on the activity of PLC-PKC signaling (Zhu et al., 2017). On the other hand, formin regulates contractility in preimplantation morphogenesis (Özgüç et al., 2022). The actin nucleator ARP2/3 is critical for blastomere cytokinesis, and its inhibition leads to the failure of blastocyst formation. The RHO-associated coiled-coil-containing protein kinase (ROCK), associated with cell migration and adhesion, vesicular trafficking, and cytoskeletal dynamics, is involved in apicobasal cell polarity proteins maintenance (Marikawa and Alarcon, 2018), and the regulation of angiomotin (AMOT) localization (Mihajlović and Bruce, 2016). AMOT is a scaffolding protein involved in cell-cell junctions. As an activator of the Hippo pathway, it is crucial for the specification of TE and ICM cells in the mouse blastocysts (reviewed in: Mihajlović and Bruce, 2017). Interestingly, recently published data on mouse and human embryos indicate that there is a tight link between actomyosin contractility, lamin-A, a component of the nuclear lamina, and AMOT stability (Skory et al., 2023). Nuclear lamina is linked to the blastomere cortex via an F-actin network. As actomyosin contractility increases during embryo development, lamin-A levels rise as well. However, in cells that underwent internalization in compacted embryos and lost their apical, actomyosin-rich domain, lamin-A becomes downregulated. This leads to the relocalization of actin nucleators from the nucleus to the cytoplasm and an increase in cytoplasmic F-actin. In consequence, AMOT is stabilized and YAP, a key transcription coregulator involved in cell lineage differentiation, undergoes phosphorylation (Hippo pathway activated). Active Hippo pathway directs inner cells towards the ICM fate. By contrast, in outer cells, lamin-A levels are upregulated. This destabilizes AMOT and prevents YAP phosphorylation (Hippo pathway inactive), promoting TE cell fate (Skory et al., 2023).

A critical and final event in preimplantation development is the formation of a blastocyst cavity, in which the radial symmetry of the embryo is broken. In mouse, the apicobasal polarity of outer TE permits the formation of an osmotic gradient that draws water from the external environment via the apical compartment of blastomeres (Eckert et al., 2004; Madan et al., 2007; Moriwaki et al., 2007) into the basal intercellular regions sealed by tight junctions (Zenker et al., 2018). Such fluid accumulation is driven by the secretion of cytoplasmic actin-coated vesicles into the intercellular space (Ryan et al., 2019). Hundreds of microlumens form throughout the mouse embryo between cell-cell contacts by hydraulic fracturing, directed by cadherin reorganization (Dumortier et al., 2019). Some microlumens also display enrichment of the apical marker, phosphorylated ezrin-radixin-moesin complex (Ryan et al., 2019). Microlumens show a swelling phase followed by a siphoning of all the fluid to a single cavity, guided by actomyosin contractions (Dumortier et al., 2019; Schliffka et al., 2023).

Existing data clearly indicate that TE functionality depends on the quality of its cytoskeleton and intracellular junctions that determine the epithelial character of this layer. Additionally, the expression and activity of proteins transporting ions, thus allowing for osmotic gradient formation and consequent cavitation (Bazer et al., 2009; Posfai et al., 2019), also play an important role in TE functioning. These factors are associated with the biomechanical properties of TE cells. It has been shown that inhibition of Na^+^/K^+^ pumps or claudins in tight junctions affects the CT of TE cells (Chan et al., 2019). Decreased TE cell tension also coincides with the disassembly of vinculin from tight junctions and disrupts tight junction seal integrity (Chan et al., 2019). Vinculin has been shown to regulate traction force transmission via myosin contractility-dependent adhesion (Dumbauld et al., 2013). Additionally, actin filament remodeling, required to form a sealed TE epithelium, is tension-sensitive (Zenker et al., 2018). Interestingly, it has been shown in mouse embryos that mechanical stretching, typical for TE cells during cavity expansion, facilitates *Cdx2* expression (Watanabe et al., 2017). *Cdx2* expression is a prerequisite for TE function (although not always for early stages of TE differentiation) in mice, humans, and domestic animals (reviewed in: Piliszek and Madeja, 2018; Posfai et al., 2019). Notably, keratins have been recently proven to be another regulator of TE fate in both mouse and human embryos: they enhance apical polarity and *Cdx2* expression in outer cells (Lim et al., 2020). Although keratin knockouts display trophoblast fragility, placental bleeding, and lethality after implantation (Baribault et al., 1993; Hesse et al., 2000; Tamai et al., 2000), depletion of keratins 8 and/or 18 (*i.e*., variants that are the most abundant in preimplantation embryos; Lu et al., 2005) does not lead to severe phenotypes up to blastocyst stage, either in mice or in cattle (Goossens et al., 2010; Lim et al., 2020). Interestingly, in mouse embryos, keratin 8/18-knockdown cells display a reduced nuclear expression of YAP (required for *Cdx2* transcription) and CDX2 itself in TE cells.

Immediately prior to implantation, blastocysts hatch from the *zona pellucida*, exposing the TE, which attaches to the endometrial epithelium of the uterus. Blastocyst attachment initiates a complex cascade of events that lead to the implantation and development of a placenta**.** Importantly, implantation requires functional TE (Bazer et al., 2009; Aplin and Ruane, 2017; Posfai et al., 2019) and failure in implantation is the main source of reproduction loss in mammals, including humans and cattle (Aplin and Ruane, 2017; D’Occhio et al., 2020).

In summary, it is clear that the biomechanical properties of embryos are highly associated with their developmental potential. By gaining insights into how biomechanical factors influence the formation and quality of embryos, and their subsequent implantation, researchers can set forth noninvasive and robust methods of assessing such properties which could help ART practitioners make more informed decisions when selecting embryos for transfer.

## 5 Quality assessment based on embryo biomechanical properties

Time-lapse recordings used for classical morphogenetic analysis of preimplantation embryos, if only covering cavitation and blastocysts expansion events, may be applied for the assessment of the biomechanical properties of TE. Mouse blastocysts with inhibited actomyosin contractility, Na^+^/K^+^ pumps, or perturbed tight junctions displayed a slower expansion rate (Chan et al., 2019). Moreover, it has been shown in mouse embryos that experimentally reduced cavity size and hydraulic pressure inside the cavity are associated with the increased number of ICM cells and perturbed specification and spatial separation of ICM lineages (EPI and PE; Chan et al., 2019; Ryan et al., 2019). Analysis of the extent of blastocyst expansion has been shown to be a predictor of pregnancy success in human embryos (Du et al., 2016), and expansion kinetics have been related to a human embryo’s ploidy: euploid embryos expanded faster than aneuploid embryos (Huang et al., 2019). The rate of blastocoel re-expansion in frozen-thawed embryos also has been associated with pregnancy likelihood in both humans and domestic animals (Leoni et al., 2008; Yin et al., 2016; Ebner et al., 2017; Lin et al., 2017; Zhao et al., 2019). Interestingly, the rate of human blastocyst re-expansion correlates with the number of TE cells (Iwasawa et al., 2019). This observation has also been confirmed in mouse embryos: smaller blastocyst size is associated with slower blastocoel expansion (Chan et al., 2019).

**FIGURE 4:**
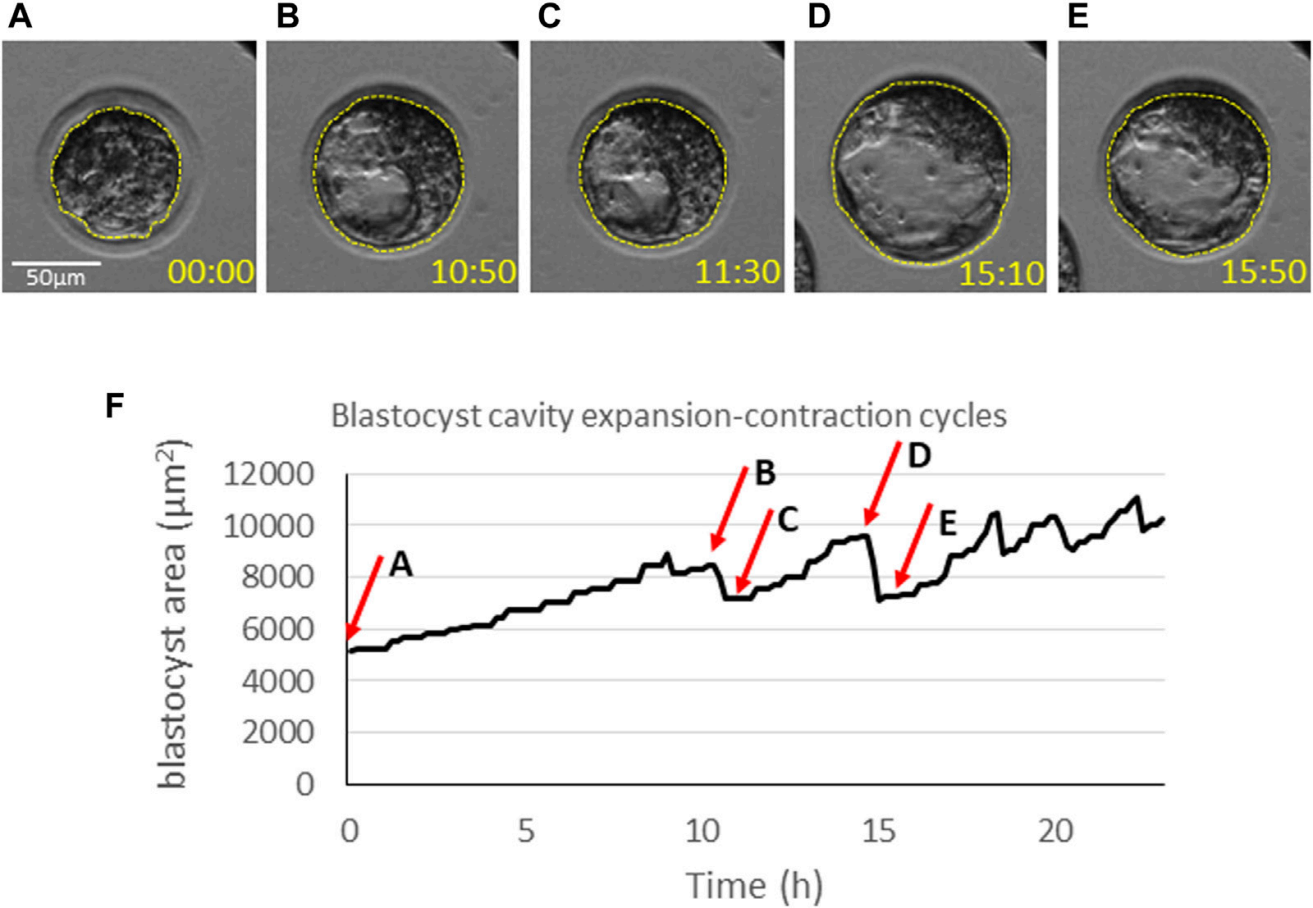
Dynamics of blastocyst cavitation. The equatorial area of blastocysts (dashed line) is measured at **(A)** the onset of cavitation, **(B)** the time-point of maximum expansion just before the first contraction, **(C)** the last phase of the first contraction, **(D)** the time-point of maximum expansion before the next contraction, **(E)** the last phase of the contraction. Time points (hh:mm) indicate the time of imaging and correspond to the graph below **(F)**. **(F)** A graph representing oscillations of the blastocyst size over time. The arrows indicate the time points corresponding to the measurements in **(A–E)**.

Blastocyst cavity volume tends to oscillate during the expansion period, undergoing contraction-expansion cycles (Figure 4). This feature seems to be an intrinsic property of the blastocysts in all animals examined, including humans (Niimura, 2003; Huang et al., 2016), and is associated with waves of mitotic divisions in TE and increasing TE cortical tension (Chan et al., 2019). However, the interpretation of these contraction-expansion cycles in terms of embryo quality is ambiguous. It has been reported that human blastocysts that transiently collapsed have less potential to give rise to pregnancy (Marcos et al., 2015; Gazzo et al., 2020; Sciorio et al., 2020) and are characterized by higher aneuploidy odds (Gazzo et al., 2020). Others have claimed that the occurrence of blastocyst collapse is not an independent predictor of reduced live birth rate (Bodri et al., 2016). It has been shown that mouse blastocysts exhibiting stronger contractions of the lumen are less likely to hatch (Shimoda et al., 2016). Moreover, unusually frequent blastocoel contractions in mouse embryos can be caused by aberrant intracellular junctions in TE (Togashi et al., 2015). While the interpretation of contraction-expansion cycles in blastocysts remains inconclusive, monitoring cavitation dynamics might provide useful insights into the quality of blastocyst cytoskeleton as well as the functionality of intracellular junctions and proteins involved in filling blastocoel with fluid. Further research is essential to validate the efficacy of such an approach and its potential for application in ART.

PIV-based analysis of cytoplasmic movement in blastomeres is yet another method to provide insights into the biomechanical properties of embryos. Cytoplasmic motion reflects the reorganization of cytoskeletal elements that are key for the movement of signaling vesicles, and, as described above, for both compaction and cavitation (Coumailleau et al., 2009; Derivery et al., 2015; Zenker et al., 2017). The reorganization of the actinomyosin cytoskeleton can be observed with PIV analysis (Özgüç et al., 2022), and holds promise as a means to monitor and assess the dynamics of these crucial phases in preimplantation embryo development. Additionally, the analysis of cytoplasmic velocity with the PIV algorithm could be applied in TE cells (Figure 1), where it is associated with the functionality of keratin cytoskeleton, crucial for subsequent implantation: keratin-depleted TE cells have a more mobile (less “rigid”) cytoplasm (Lim et al., 2020).

Keratin 8/18 depletion also increased the apical curvature of TE cells, which is indicative of lower apical tension (Lim et al., 2020). Therefore, microaspiration or indentation methods may help in detecting embryos with keratin defects. Interestingly, in mice, CT of TE cells is associated with the embryo size at least at the early blastocyst stage: smaller embryos (obtained by dissection of the whole embryo in halves or quarters) display higher CT (Chan et al., 2019). Therefore, measuring the CT of cells could provide information on the functionality of the cellular cytoskeleton, intracellular junctions, and ion pumps required for cavitation in TE cells (Chan et al., 2019; Lim et al., 2020)**.**

## 6 Summary

This review offers a comprehensive overview of the molecular mechanisms that underlie the biomechanical properties of oocytes and embryos, along with the potential noninvasive techniques for assessing those properties. Our intent is to bridge the gap between scientific research and practical applications, providing a background for the suitability of the proposed techniques in the context of ART.

As highlighted here, cytoskeletal proteins play a pivotal role in determining the developmental potential of oocytes and embryos. Cytoskeleton, particularly its actomyosin component, governs key intracellular processes such as cell cycle progression or organelle trafficking, and intercellular processes such as compaction and cavitation. Elucidating biomechanical biomarkers characterizing a high-quality oocyte and properly developing preimplantation embryo with functional TE could provide a novel approach for evaluating quality beyond conventional morphological assessment. We draw attention to promising techniques, such as analysis of cytoplasmic movement or cavitation-related morphokinetic parameters as well as cortical tension and elasticity measurements, which may offer novel insights into oocyte and embryo viability.

The integration of biomechanical assessments into ART could refine currently used procedures. Conventional methods of oocyte and embryo evaluation based solely on morphological criteria have limitations in predicting implantation and pregnancy rates accurately. Biomechanical assessment provides a more comprehensive understanding of oocyte and embryo quality, potentially enhancing the selection of oocytes and embryos with higher developmental potential. While some questions and ambiguities persist, and further research and validation are imperative to establish the reliability and effectiveness of these techniques in a clinical setting, ongoing research in cellular biomechanics holds great potential for enhancing the success rates of fertility treatments.

## Abbreviations

ART, assisted reproductive technologies; CT, cortical tension; EPI, epiblast; HGM, harmonic generation microscopy, ICM, inner cell mass; IVF, *in vitro* fertilization; OCM, optical coherence microscopy; PE, primitive endoderm; PIV, particle image velocimetry; PLM, polarized light microscopy; QPI, quantitative phase imaging; ROS, reactive oxygen species; TE, trophectoderm.
